# Barriers and Opportunities to Advancing Women in Leadership Roles in Vector Control: Perspectives from a Stakeholder Survey

**DOI:** 10.4269/ajtmh.17-0693

**Published:** 2018-03-19

**Authors:** Mary H. Hayden, Erika Barrett, Guyah Bernard, Eunice N. Toko, Maurice Agawo, Amanda M. Okello, Jayleen K. L. Gunn, Kacey C. Ernst

**Affiliations:** 1 Research Applications Laboratory, National Center for Atmospheric Research, Boulder, Colorado;; 2 Mel and Enid Zuckerman College of Public Health, University of Arizona, Tucson, Arizona;; 3 Department of Biomedical Sciences and Technology, School of Public Health and Community Development, Maseno University, Maseno, Nyanza, Kenya

## Abstract

Increasing the active participation of professional women in vector control (VC) activities may help promote greater gender equity in the workplace and reduce the burden of vector-borne diseases. This stakeholder survey examined the current roles and perspective of professionals employed in the VC sector in Kenya, Indonesia, India, and other countries. The largest barriers that women face in pursuing leadership roles in the VC sector include lack of awareness of career opportunities, limitations based on cultural norms, and the belief that VC is men’s work. These barriers could be addressed through improving education and recruitment campaigns, as well as supporting higher education and mentoring programs. Females were almost six times more likely to be encouraged to pursue leadership positions in their organization compared with male respondents (odds ratio = 5.9, *P* > 0.03, 95% confidence interval: 1.19, 29.42). These findings suggest that once women are recruited into the VC workforce, they face minimal discrimination and have increased leadership opportunities.

Vector-borne diseases (VBDs) continue to increase in scale and intensity. Despite concerted efforts to reduce their burden, in 2016, VBDs accounted for more than 17% of all infectious diseases and more than 1 million deaths annually.^1^ Achieving widespread, consistent adoption of interventions to reduce vector abundance continues to be an obstacle to decreasing the disease burden in many parts of the world; increasing participation of women and promoting them into leadership roles may enhance the uptake of interventions.^2–5^ Examining VBD risk prevention and control strategies through a gender lens has been suggested by professionals as a means to increase the efficiency, effectiveness, and sustainability of vector control (VC).^5,6^ Despite the global impact of VBDs on all population segments, historically, men have taken leadership roles in the uptake and execution of VC measures.^7^ However, women are often key agents of change in programs to combat public health challenges, including VBDs.^8,9^ Advancing women’s involvement in VC and promoting gender equity in the workplace may result in better control of VBDs.^10^ The purpose of this study was to examine the current roles and perspectives of professionals employed in VC and identify potential strategies that will increase leadership roles for women.

As part of our larger study, we conducted focus group discussions (FGDs) and key informant interviews (KIIs) with stakeholders (e.g., decision-makers, those involved in VC activities or management, and health community leaders) in Indonesia and Kenya.^11^ We compiled an expanded list of stakeholders and their contact information, from our FGDs and KIIs, through personal connections from other countries, and from a review of reports and literature. A cross-sectional stakeholder survey (SS) was then developed through the online survey software Qualtrics.^12^ The SS included 30 questions (i.e., binary, multiple choice, multiple answer, free text, and Likert scale) derived from initial group discussions and interviews. The survey was active from August 18, 2016 to November 30, 2016, and distributed using the Dillman tailored design method^13^ via e-mail through Qualtrics.^12^ At the end of the SS, participants could identify other colleagues who should take the survey, also known as “snowball sampling.” The SS was distributed to individuals identified through snowball sampling within 48 hours of their nomination following the same method. In addition, the survey was sent out to one listserv by a key stakeholder in Kenya and to two larger listservs provided later in the data collection process by stakeholders in Indonesia and India.

Respondents were excluded if they indicated their organization was not involved in VC or completed less than 10% of the SS. Descriptive analyses of respondent and organization demographics and reported barriers and opportunities for increasing women’s participation in VC were completed using STATA (Stata Corp., College Station, TX).^14^ Kruskal–Wallis H tests were used to determine differences between perceived gender associated with specific VC activities on a scale of Male, Somewhat Male, Neutral, Somewhat Female, and Female overall and stratified by region and gender. We interpreted the results in terms of means instead of medians because the groups for country and gender did not have similarly shaped distributions. Finally, we conducted logistic regression analyses stratified by the gender of respondents to assess differences in gender discrimination questions. The study protocol was reviewed and approved by the Human Subjects Committee at the National Center for Atmospheric Research.

Of the 252 total surveys that were distributed through e-mail (63 through snowball sampling), 93 were included in the final analysis for a response rate of 38%. Of the 93 respondents, 32% were from Indonesia, 26.7% from Kenya, 18.7% from India, and 22.6% from other regions. Demographic data were missing for 18 respondents. Respondents were gender-balanced, mainly older than 40 years of age, married, with postsecondary education, and had at least one child at home (Table 1). Respondents came from academia or research, state governments, and nonprofit organizations, and had been at their organization on an average of 12 years, typically supervising on average 10 males and eight females (Table 1).

**Table 1:** Basic demographics and organization characteristics of participating respondents

Basic demographics	Total *N* (%)	Male *N* (%)	Female *N* (%)
Age in years			
20–39	30 (40.0)	18 (47.4)	12 (32.4)
40–59	40 (53.3)	19 (50.0)	21 (56.8)
60+	5 (6.7)	1 (2.6)	4 (10.8)
Marital status			
Married or living with partner	61 (81.3)	26 (68.4)	35 (94.6)
Single	9 (12.0)	7 (18.4)	2 (5.4)
Divorced, separated, or widow	5 (6.7)	5 (13.2)	0 (0)
Children in the home	54 (58.1)	23 (42.6)	31 (57.4)
Education			
Less than high school	1 (1.3)	0 (0)	1 (2.7)
Diploma	1 (1.3)	1 (2.6)	0 (0)
Bachelor’s degree	9 (12.0)	6 (15.8)	3 (8.1)
Master’s degree	27 (36.0)	10 (26.3)	17 (46.0)
Doctorate	37 (49.4)	21 (55.3)	16 (43.2)
Country			
Kenya	21 (25.0)	12 (31.6)	8 (21.6)
Indonesia	28 (33.3)	10 (26.3)	14 (35.1)
India	21 (25.0)	2 (5.3)	12 (32.5)
Other	14 (16.7)	14 (36.8)	3 (10.8)
Organization			
Federal government	9 (9.8)	6 (15.8)	3 (8.1)
State government	18 (19.5)	5 (13.2)	9 (24.3)
Local government	11 (12.0)	1 (2.6)	5 (13.6)
Private/for-profit or environmental	11 (12.0)	8 (21.0)	2 (5.4)
Nonprofit	16 (17.4)	6 (15.8)	6 (16.2)
Academic/research	21 (22.8)	10 (26.3)	10 (27.0)
Other	6 (6.5)	2 (5.3)	2 (5.4)
Activities (multiple select)			
Community engagement and education	56	29 (51.8)	27 (48.2)
Research	55	33 (60.0)	22 (40.0)
Vector surveillance	49	25 (51.0)	24 (49.0)
Spraying and environmental management	37	15 (40.5)	22 (59.5)
Larviciding and distribution of *Gambusia*	30	13 (43.3)	17 (56.7)
Selling and marketing PPE and products	9	6 (66.66)	3 (33.33)

PPE = personal protective equipment.

Overall, the response rate for the SS was 38%; however, only 30 of the 252 (11.9%) surveys sent out from the initial stakeholder list were completed. This suggests the importance of snowball sampling and the benefits of providing respondents the option of sharing the survey with peers and colleagues. Face-to-face recruitment conducted in Kenya enhanced participation rates from this country, yielding an 84% response rate in Kenya compared with 20% response rate in Indonesia, excluding snowball sampling.

The perspectives of study participants indicated low levels of perceived gender discrimination in the workplace. Three-quarters (75%) felt respected in their workplace (no differences by gender) and would recommend working at their workplace to a close female friend, colleague, or family member (80%). Most (70%) were aware of government programs to increase women’s participation in VC, but only 43% felt the programs were effective or extremely effective.

A few respondents (five males and three females) noted instances of gender discrimination in the workplace, including gender bias during selection of jobs and less respect for female coworkers. Just under half (46%) were aware of policies/training in their workplace on gender discrimination; however, only 38% of those felt such training worked well or extremely well. There were no differences by country or gender. Female respondents were 5.9 times more likely to report being encouraged to pursue leadership positions in their organization than males (OR = 5.9, *P* = 0.03, 95% confidence interval: 1.19, 29.42).

In general, governmental organizations participated in all VC activities except the selling and marketing of personal protective equipment (PPE). Nonprofit organizations contributed the most to community engagement and education, and academic and research organizations were more involved in research, vector surveillance, and community engagement activities. Lack of awareness of career opportunities, cultural norms, the belief that VC is men’s work, household obligations, and lack of job security during pregnancy were the most frequently reported barriers women face to entering the field of VC (Figure 1A). The strategies considered most effective to increasing women’s participation were making structural changes to facilities, providing gender-specific protective equipment, ensuring job security during pregnancy, and providing micro-finance to start VC businesses. However, few of these strategies had been implemented previously by the organizations (Figure 1B).

**Figure 1.**
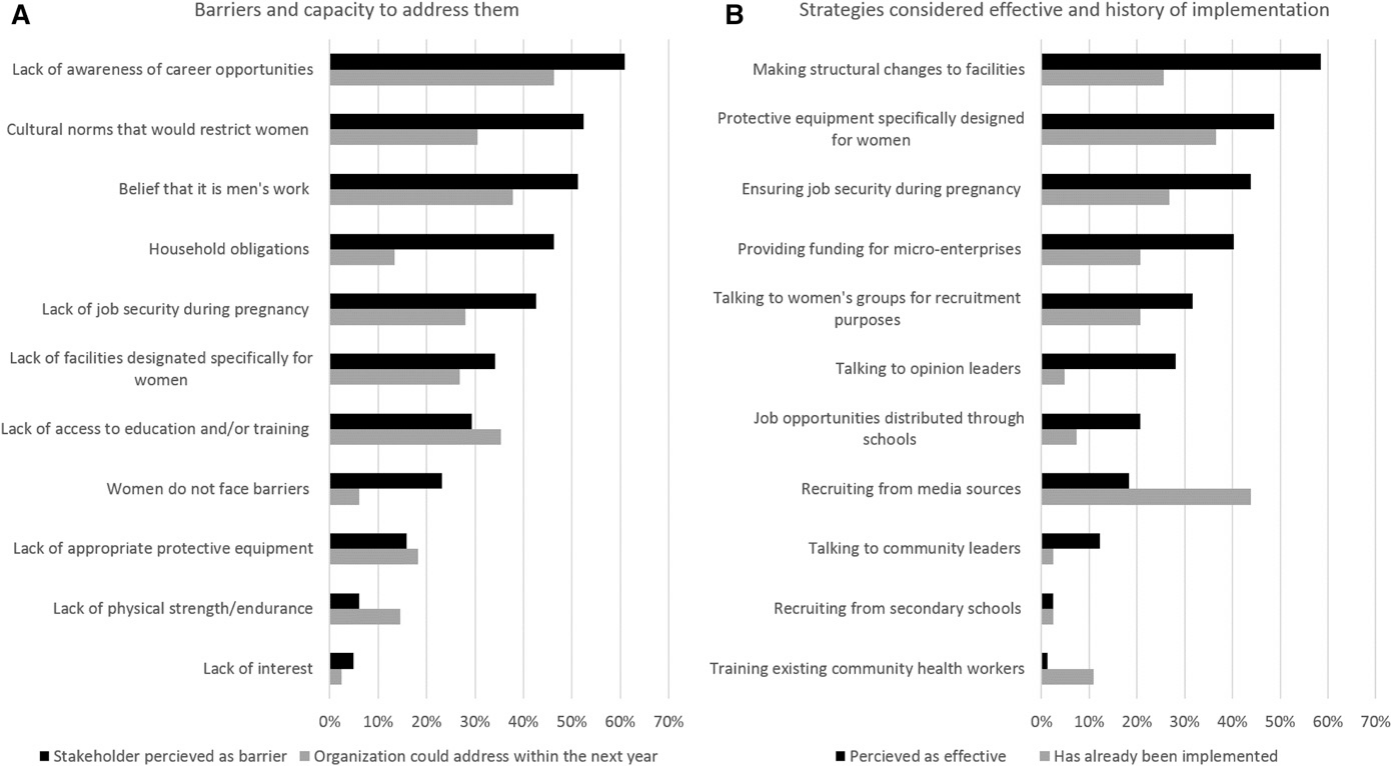
(**A**) Proportion of stakeholders indicating a proposed barrier to women being employed in vector control (VC) is important and whether they can address it within their organization in the next year. (**B**) Proportion of stakeholders who perceive the proposed strategy to be effective in increasing the number of women in VC and the proportion whose organization has implemented the said strategy previously.

The most frequently reported barrier to women engaging in VC was the lack of awareness of opportunities (Figure 1A). When examining the history of strategies used in respondent organizations to recruit women into VC positions, the most commonly reported strategy was mass media. More community-based efforts, such as working with women’s groups, schools, and opinion leaders, were rarely used, despite a higher proportion of respondents indicating they would be effective (Figure 1B). Changing recruitment strategies may yield an effective increase in women’s participation.

Most respondents indicated that applying pesticides, participating in vector collection, and traveling for work were mostly male VC activities whereas selling PPE, and education and collaboration were mostly female VC activities (Figure 2). A Kruskal–Wallis H test was conducted to determine if rating-scale questions were different for the three countries (Kenya, Indonesia, and India) and for differences in gender. Results showed that there was a statistically significant difference in the ranking of activities as mostly male to mostly female among countries for applying pesticides (χ^2^[3] = 10.05, *P* = 0.02) with India having a higher proportion ranking this as a male activity and no significant differences for gender.

**Figure 2.**
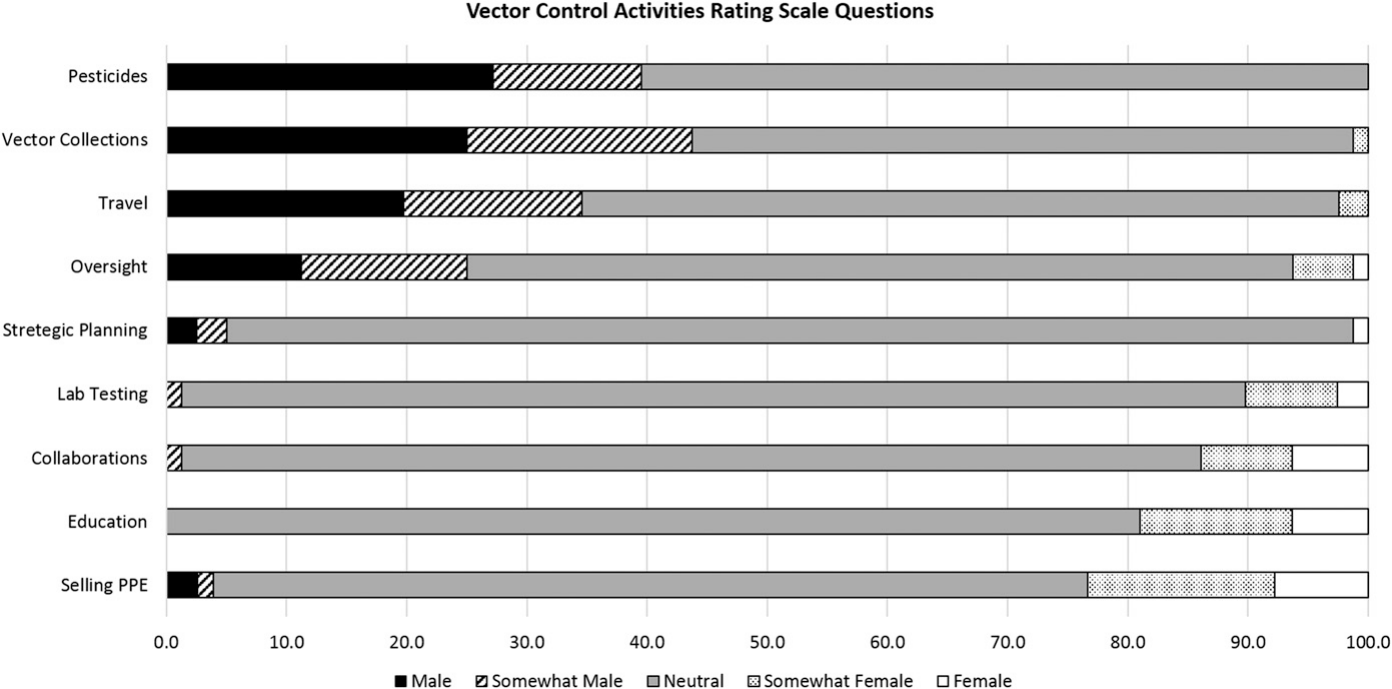
Vector control activities rating-scale questions.

Respondents also indicated that changes to the physical space and equipment used would be highly effective strategies to recruit women into VC positions. The African Indoor Residual Spray (AIRS) program has recognized the importance of having separate facilities and specific equipment for spray operators that are designed to fit women.^15^ The multipronged focus on gender in specific countries by AIRS has resulted in increased participation by women. Not all VC will require physical space for women or tailored safety equipment; primarily this will be important in programs that emphasize the use of pesticides. Programs that use pesticides also need to address alternative opportunities for women during pregnancy as the AIRS program has.

Pesticide application was primarily associated with the male gender; however, conducting vector collections for surveillance, traveling from home, and needing to be away from home overnight were also indicated as more “male.” This may be associated with more traditional gender norms in which women are expected to be at home with children, preparing meals, and not associating with unknown persons. This is supported by the respondent perception that cultural norms are a leading barrier to women engaging in VC activities.

Although limited evidence suggests that women are not well incorporated into VC programs, this study suggests that when they are in leadership roles, they face minimal discrimination and are encouraged to take on leadership positions. Once women are employed in the VC field, they may be encouraged to pursue more leadership positions to equalize gender differences throughout all organizational levels. The individuals included in this work, however, are predominantly very well educated, with 85% having a master’s degree or higher. A substantial proportion of the VC positions does not require higher education, and it has been demonstrated that higher education is associated with more equitable gender norms.^16,17^ Furthermore, roles such as indoor residual spray operators, community organizers, vector habitat reduction teams, and education and outreach leaders require a greater interface with communities that may be more likely to hold to traditional gender norms. Follow-up studies on the influence of gender norms on engagement in VC programs at a community level are underway.

## Supplementary Material

Supplemental Questionnaire.
