# Supplementary material for: Barriers and Opportunities to Advancing Women in Leadership Roles in Vector Control: Perspectives from a Stakeholder Survey
Source: Am J Trop Med Hyg. 2018 Mar 19;98(5):1224–7. doi: 10.4269/ajtmh.17-0693 (PMC5953369; doi:10.4269/ajtmh.17-0693)
Supplement: Supplementary file 1 [file tpmd170693.SD1.pdf]

Women in Vector Control: Stakeholder Survey

Bahasa version of survey available upon request

Start of Block: Default Question Block

X→ X→

The National Center for Atmospheric Research and the University of Arizona are collaborating on a project funded by the Bill and Melinda Gates Foundation that examines the current roles and perceptions of women in vector control, and seeks to identify potential strategies that will accelerate women's involvement in vector control. You have been contacted to participate in this survey given your expertise in and knowledge of vector-borne diseases. Your participation is essential to determine next steps in involving more women in vector control to help reduce the burden of vector-borne diseases. The survey contains approximately 30 short questions and will take between 10-15 minutes to complete. Demographic information will be utilized for research purposes only. No individual information will be reported. The results will be disseminated through a publication and/or stakeholder meeting slated for late 2016/early 2017. CONFIDENTIALITY: Individual responses will not be reported. All responses that you provide will remain completely confidential. Quotations in the comment box may be used in their entirety if they do not compromise confidentiality. Do you want to proceed?

O Yes (1)

O No (0)

Skip To: textno If = No (0)

---

text1 We'd like to start by asking you some questions about your organization and the programs in which you're involved.

X→ X→

org Is your organization involved in the prevention and control of pathogens transmitted by insects, ticks, or mites?

- O Yes (1)
- O No (0)
- O Don't know (3)

Skip To: textno If org = No (0)

Skip To: textno If org = Don't know (3)

Skip To: org If org = Yes (1)

---

textno Thank you for your time. We are targeting our survey specifically towards people involved in vector prevention and control.

To help us ensure this is distributed to the right individuals, please indicate the full name and e-mail of any colleagues who you think should participate or would be interested in participating in this survey.

____

____

____

____

____

Skip To: End of Survey If

---

org What type of organization do you represent?

- O Federal government (1)
- O State government (2)
- O County government (3)
- O City government (4)
- O Private/For-profit organization (5)
- O Non-profit organization (6)
- O Environmental organization (7)
- O Academic/research institution (8)
- O Hospital/medical institution (9)
- O Other (Please specify) (10) ____________________

orgdis On what disease(s) does your organization focus? Please check all that apply:

- ▢ Malaria (1)
- ▢ Aedes transmitted viruses such as dengue, chikungunya, Zika and/or yellow fever (2)
- ▢ Filariasis (3)
- ▢ Rift Valley Fever (4)
- ▢ Encephalitis (5)
- ▢ Plague (6)
- ▢ Tick-borne diseases (Please specify) (7) ____
- ▢ Onchoceriasis (8)
- ▢ Trypanosomiasis (9)
- ▢ Leishmaniasis (11)
- ▢ Other (10) ____

orgactivity What types of activities best describe your organization's involvement with vector control and vector-borne diseases? Please check all that apply:

- ▢ Coordination of prevention and control activities among community organizations (1)
- ▢ Community engagement and organization (2)
- ▢ Community clean-up of trash/vector habitat (3)
- ▢ Education and outreach (4)
- ▢ Development of materials for education and outreach (5)
- ▢ Environmental management such as sanitation, drainage of ditches and swamps (6)
- ▢ Mass distribution of bed-nets (7)
- ▢ Indoor Residual Spray (IRS) (8)
- ▢ Space spraying indoors (9)
- ▢ Outdoor residual spraying (10)
- ▢ ULV spraying (fogging) (11)
- ▢ Aerial spraying (12)
- ▢ Larviciding (13)
- ▢ Distribution of Gambusia (14)
- ▢ Selling personal protective products such repellents (15)
- ▢ Selling commercial/industrial use insecticides (19)
- ▢ Marketing protective products such as mosquito coils, in-room spatial repellents/plug-ins (16)
- ▢ Vector surveillance and monitoring (17)

▢ Research (20)

▢ Other (Please specify) (18) ____

---

years How many years have you been with this organization?

▼ Under 1 year (0) ... 30 (30)

---

malesup How many male employees do you supervise (directly report to you)?

▼ 0 (0) ... 50+ (50)

---

femalesup How many female employees do you supervise (directly report to you)?

▼ 0 (0) ... 50+ (50)

---

supmf Is your direct supervisor male or female?

O Male (1)

O Female (0)

---

citycountry In what city/country do you work?

- O City (1) ____
- O Country (2) ____

text2 Next, we now want to ask you a few questions about your experiences in your workplace as a vector-bone disease professional. We are interested in learning more about the roles and equity in your workplace, so please refer to the definitions below when answering the following questions.   The aim of **gender equity** is to achieve broadly equal outcomes for people in the workplace. Gender equity is achieved when people are able to access and enjoy the same rewards, resources, and opportunities regardless of gender. This can include equal pay, removal of barriers to full and equal participation in the workplace, access to all occupations, and elimination of discrimination.   **Gender discrimination** in the workplace involves treating someone unfavorably because of the person’s sex, whether he/she is applying for a job or is a current employee. This can include hiring, firing or promotions, pay discrepancies, job mis-classifications, and benefit discrepancies, all based on the acceptability of the person’s gender.

X→ X→

govprog Are you aware of government programs in your country to achieve gender equity?

- O Yes (1)
- O No (0)
- O Unsure (3)

Skip To: QID57 If govprog = No (0)

Skip To: QID57 If govprog = Unsure (3)

X→ X→

progeff
How effective are your country's programs to achieve gender equity?

- O Extremely effective (5)
- O Effective (4)
- O Neither effective nor ineffective (3)
- O Ineffective (2)
- O Extremely ineffective (1)

Next, we now want to ask you a few questions about your experiences in your workplace as a vector-bone disease professional.

X→ X→

leadposit Are you encouraged to pursue greater leadership positions within your workplace?

- O Yes (1)
- O No (0)
- O Unsure (3)

X→ X→

respect Do you feel respected in the workplace?

- O Yes (1)
- O No (0)
- O Unsure (3)

---

genderdis Have you experienced and/or witnessed any gender discrimination against females during the hiring process and/or within the workplace?

- O Yes (1)
- O No (0)
- O Unsure (3)

Skip To: training If genderdis = No (0)

Skip To: training If genderdis = Unsure (3)

---

genderdistext Please explain the discrimination against females in the workplace that you have witnessed.

____

____

____

____

____

---

training Does your organization have special trainings and policies in place to protect women from discrimination?

O Yes (1)

O No (0)

O Unsure (3)

Skip To: suggdes If training = No (0)

Skip To: suggdes If training = Unsure (3)

---

policy How well does your organization enforce these policies?

O Extremely well (5)

O Well (4)

O Neither well nor poorly (3)

O Poorly (2)

O Not at all (1)

---

suggdes How well are your suggestions and decisions considered or implemented by your organization?

- O Extremely well (5)
- O Well (4)
- O Neither well nor poorly (3)
- O Poorly (2)
- O Not at all (1)

X→ X→

recpeer Would you recommend working for your organization to a close personal female contact (e.g. relative, friend, colleague)?

- O Yes (1)
- O No (0)
- O Unsure (3)

X→ X→

eqopp In which of the following do you believe males and females have equal opportunities to engage in vector control? Please check all that apply:

- ▢ In academia (1)
- ▢ In a professional capacity such as working for a mosquito abatement district (2)
- ▢ At the community level (3)
- ▢ In government positions (4)
- ▢ In industry (7)
- ▢ Non-Governmental Organizations (NGOs) (8)
- ▢ No where (0)
- ▢ Other (Please specify) (5) ____

mef Please indicate whether or not you feel a specific vector control activity is better for men (male or somewhat male) , better for women (female or somewhat female) or equally suitable for both genders (equal)?

	Male (0)	Somewhat Male (1)	Equal (2)	Somewhat Female (3)	Female (4)
Applying and handling pesticides (pesticides)	O	O	O	O	O
Driving motorbikes and vehicles within and between communities (travel)	O	O	O	O	O
Oversight of field teams conducting vector control activies that are predominantly or completely male (oversight)	O	O	O	O	O
Conducting entomological collections including overnight adult landing collection (collections)	O	O	O	O	O
Selling personal vector control products within a community (selling ppe)	O	O	O	O	O
Building collaborations with community partners	O	O	O	O	O

(collab)					
Educating the community about vector control strategies (education)	O	O	O	O	O
Developing strategic plans for vector control programs (mef_11)	O	O	O	O	O
Laboratory testing and maintaining colonies (mef_12)	O	O	O	O	O

barriers What barriers do women face while trying to pursue opportunities in vector control? Please check all that apply:

- ▢ Lack of awareness of career opportunities in vector control (2)
- ▢ Lack of access to education and/or training (3)
- ▢ Belief that it is men's work (4)
- ▢ Cultural norms that would restrict women (such as working on mixed gender teams, traveling outside the community, entering men's homes) (5)
- ▢ Household obligations (6)
- ▢ Lack of appropriate protective equipment (7)
- ▢ Lack of job security during pregnancy (8)
- ▢ Lack of facilities designated specifically for women, such as bathrooms, changing rooms, showers (9)
- ▢ Lack of physical strength/endurance (12)
- ▢ Lack of interest (13)
- ▢ Women do not face barriers (10)
- ▢ Other (Please specify) (11) ____

barriersinc Which of these barriers could your organization address within the next year to help increase the number of women in vector control? Please check all that apply:

- ▢ Lack of awareness of career opportunities in vector control (2)
- ▢ Lack of access to education and/or training (3)
- ▢ Belief that it is men's work (4)
- ▢ Cultural norms that would restrict women (such as working on mixed gender teams, traveling outside the community, entering men's homes) (5)
- ▢ Household obligations (6)
- ▢ Lack of appropriate protective equipment (7)
- ▢ Lack of job security during pregnancy (8)
- ▢ Lack of facilities designated specifically for women, such as bathrooms, changing rooms, showers (9)
- ▢ Lack of physical strength/endurance (12)
- ▢ Lack of interest (13)
- ▢ Women do not face barriers (10)
- ▢ Other (Please specify) (11) ____

extraeff Does your organization make extra efforts to recruit women and/or promote greater involvement of women in vector control, such as through gender quotas (quotas that are designed to create equal representation among genders) specialized recruitment, modification of facilities, provision of specific equipment, or training programs?

- Yes (1)
- No (0)
- Unsure (3)

stratcb Which strategies would best increase women's participation in **community-based** vector control? By community-based we mean vector-control that is organized and takes place at the community level. Please check all that apply:

- ▢ Making structural changes to facilities such as adding showers, changing rooms and bathrooms to accommodate women (22)
- ▢ Ensuring job security during pregnancy (23)
- ▢ Ensuring availability of protective equipment specifically designed for women (24)
- ▢ Talking to women's groups for recruitment purposes (1)
- ▢ Talking to opinion leaders (such as politicians, business leaders, celebrities) for recruitment purposes (2)
- ▢ Talking to community leaders (including religious leaders, chiefs, elders) for recruitment purposes (3)
- ▢ Recruiting from media sources (such as radio, television, newspapers) (4)
- ▢ Sending fliers home with children from school for open positions in vector control (5)
- ▢ Recruiting from secondary schools (6)
- ▢ Training existing community health workers (7)
- ▢ Providing funding for micro-enterprises (8)
- ▢ Other (Please specify) (9) ____
- ▢ None (10)

stratorgcb Which strategies has your organization used to increase women's participation in community-based vector control programs? Please check all that apply:

- ▢ Making structural changes to facilities such as adding showers, changing rooms and bathrooms to accommodate women (22)
- ▢ Ensuring job security during pregnancy (23)
- ▢ Ensuring availability of protective equipment specifically designed for women (24)
- ▢ Talking to women's groups for recruitment purposes (1)
- ▢ Talking to opinion leaders (such as politicians, business leaders, celebrities) for recruitment purposes (2)
- ▢ Talking to community leaders (including religious leaders, chiefs, elders) for recruitment purposes (3)
- ▢ Recruiting from media sources (such as radio, television, newspapers) (4)
- ▢ Sending fliers home with children from school for open positions in vector control (5)
- ▢ Recruiting from secondary schools (6)
- ▢ Training existing community health workers (7)
- ▢ Providing funding for micro-enterprises (8)
- ▢ Other (Please specify) (9) ____
- ▢ None (10)

---

beststrat1 Which of these strategies do you think has worked best?

____

____

____

____

____

stratreg Which strategies would best increase women's participation in regional or higher leadership level vector control programs? Please check all that apply:

- ▢ Higher education scholarships (1)
- ▢ Women's chapters in national and international organizations (2)
- ▢ Short courses and cross-disciplinary training such as double degree programs in entomology and public health/health education (3)
- ▢ Gender quotas (4)
- ▢ Mentorship programs (5)
- ▢ International associations/societies dedicated to engaging women in vector-control (6)
- ▢ Organizational sensitivity to gender based issues (7)
- ▢ Other (Please specify) (8) ____
- ▢ None (9)

tratorgreg Which strategies has your organization used to increase women's participation in regional or higher leadership level vector control programs? Please check all that apply:

▢ Higher education scholarships (1)

▢ Women's chapters in national and international organizations (2)

▢ Short courses and cross-disciplinary training such as double degree programs in entomology and public health/health education (3)

▢ Gender quotas (4)

▢ Mentorship programs (5)

▢ International associations/societies dedicated to engaging women in vector-control (6)

▢ Organizational sensitivity to gender based issues (7)

▢ Other (Please specify) (8) ____

▢ None (9)

---

beststrat2 Which of these strategies do you think has worked best?

____

____

____

____

____

Finally, we would like to ask several demographic questions to contextualize our findings. This will also allow us to determine if opinions and experiences vary by age, gender, and family situation.

age What is your age?

- 20-30 years old (1)
- 31-40 years old (2)
- 41-50 years old (3)
- 51-60 years old (4)
- 61-70 years old (5)
- 71 + years (6)

gender What is your gender?

- Male (1)
- Female (0)

marital What is your marital status?

- O Never in union (1)
- O Married (2)
- O Single (3)
- O Living with partner (4)
- O Divorced (5)
- O Separated/no longer living together (6)
- O Widow/Widower (7)

X→ X→

children Do you have children living at home?

- O Yes (1)
- O No (0)

Skip To: education If children = No (0)

Skip To: fiveyoung If children = Yes (1)

X→ X→

fiveyoung How many children living in your home are five years of age and younger?

▼ 0 (0) ... 15+ (15)

X→ X→

fiveold How many children living in your home are older than five years of age?

▼ 0 (0) ... 15+ (15)

X→ X→

education What is the highest level of education that you have completed?

▼ Less than High School (1) ... Doctorate (7)

---

comments Please write any additional comments you may have:

____

____

____

____

____

---

othrcoll Thank you for your time in completing this survey! To help us ensure this is distributed to the right individuals, please indicate the full name and e-mail of any colleagues who you think should participate or would be interested in participating in this survey.

____

____

____

____

____

End of Block: Default Question Block
